# Mushrooms as Sustainable Protein Alternatives: Nutritional–Functional Characterization and Innovative Applications in Meat Analogs, Functional Snacks, and Beverages

**DOI:** 10.3390/foods15081301

**Published:** 2026-04-09

**Authors:** Subhash V. Pawde, Samart Sai-Ut, Passakorn Kingwascharapong, Jaksuma Pongsetkul, Shusong Wu, Jia-Qiang Huang, Zhaoxian Huang, Young Hoon Jung, Saroat Rawdkuen

**Affiliations:** 1 Unit of Innovative Food Packaging and Biomaterials, School of Agro-Industry, Mae Fah Luang University, Chiang Rai 57100, Thailand; pawdesubhash333@gmail.com; 2 Department of Food Science, Faculty of Science, Burapha University, Chonburi 20131, Thailand; 3 Department of Fishery Products, Faculty of Fisheries, Kasetsart University, Bangkok 10900, Thailand; 4 School of Animal Technology and Innovation, Institute of Agricultural Technology, Suranaree University of Technology, Nakhon Ratchasima 30000, Thailand; 5 College of Animal Science and Technology, Hunan Agricultural University, Changsha 410128, China; 6 Department of Nutrition and Health, China Agricultural University, Beijing 100083, China; 7 School of Food Science and Engineering, Hainan University, Haikou 570228, China; 8 School of Food Science and Biotechnology, Kyungpook National University, Daegu 41566, Republic of Korea

**Keywords:** mushroom, sustainability, meat analogs, future food, β-glucans

## Abstract

Global demand for sustainable protein has intensified amid environmental, public health, and ethical concerns surrounding conventional animal agriculture. Edible mushrooms have emerged as promising next-generation protein sources, delivering 19–35% protein (dry weight) with complete essential amino acid profiles and digestibility rates of 60–80%. Beyond protein, mushrooms provide bioactive compounds, including β-glucans, ergothioneine, phenolic acids, and vitamin D_2_, supporting immunomodulatory, antioxidant, and anti-inflammatory functions. Enzymatically derived bioactive peptides further demonstrate antihypertensive and antimicrobial activity. This review systematically examines mushroom protein properties, processing technologies, and product performance across three application categories: meat analogs, functional snacks, and beverages. Advanced processing technologies including high-moisture extrusion, ultrasonic-assisted extraction, and microencapsulation have improved bioactive preservation and digestibility. From an environmental perspective, mushroom cultivation requires 85–90% less water and land than animal agriculture, with 80% fewer greenhouse gas emissions. However, critical gaps remain: extraction efficiency varies 3-fold across studies, only 15–23% of commercial products are supported by clinical trials, and techno-economic analyses are largely absent. Standardized processing protocols, large-scale clinical validation, and harmonized quality standards are essential to establish mushrooms as viable, commercially scalable protein alternatives.

## 1. Introduction

The global demand for sustainable and nutritionally complete protein sources has intensified significantly in response to the growing environmental, public health, and ethical challenges associated with conventional animal agriculture. Livestock production accounts for approximately 20% of global anthropogenic greenhouse gas emissions [1] and consumes disproportionate quantities of land and freshwater resources. Concurrently, excessive consumption of red and processed meat has been independently associated with elevated risk of diet-related chronic diseases, including cardiovascular disorders, type 2 diabetes, and obesity [2]. With the global population projected to reach 9.7 billion by 2050, the development of protein systems that are capable of delivering a comparable nutritional value with substantially reduced ecological impact has become a research and industrial priority of the highest order [3].

Plant-based proteins derived from soy protein isolates, pea protein, wheat gluten, and legume concentrates currently represent the most commercially advanced alternative protein systems [4]. However, these sources are associated with persistent limitations including allergenicity concerns, incomplete or imbalanced essential amino acid profiles, the presence of antinutritional factors such as phytates and trypsin inhibitors, extensive processing requirements that conflict with consumer demand for clean-label products, and sensory challenges limiting mainstream acceptability [5,6]. These limitations have driven intensified scientific and industrial interest in novel protein sources that can address these gaps while delivering superior nutritional functionality and consumer acceptability.

Edible mushrooms have emerged as scientifically compelling candidates for sustainable protein innovation, occupying a biologically unique niche between the plant and animal kingdoms. Unlike conventional plant proteins, mushrooms provide complete essential amino acid profiles with digestibility rates of 60–80%, substantially surpassing most legumes, cereals, and other plant-based sources, and they are comparable to many animal protein benchmarks [7]. The protein content ranges from 19 to 35% on a dry weight basis, which is comparable to or exceeding that of pork, beef, and poultry, while mushrooms simultaneously deliver a distinctive bioactive compound matrix that is absent from conventional plant protein alternatives [8].

Beyond protein, mushrooms contain β-glucans characterized by β-1,3 and β-1,6 glycosidic linkages (16–35 g/100 g dry weight), providing immunomodulatory and antioxidant activities through interactions with Dectin-1 receptors and related innate immune pathways [5]. Additional bioactive constituents including phenolic compounds (gallic acid, protocatechuic acid, p-hydroxybenzoic acid), triterpenes, ergothioneine, and ergocalciferol (vitamin D_2_) contribute multi-dimensional health benefits that are directly relevant to functional food development [9]. Critically, enzymatic hydrolysis of mushroom proteins generates bioactive peptides with demonstrated antioxidant, antihypertensive, and antimicrobial activities, representing a significant yet underexplored dimension of mushroom protein value. The techno-functional properties of mushrooms, particularly their fibrous hyphal texture that closely mimics meat characteristics, superior water-holding capacity, and natural umami compounds, including glutamic acid (2.8–4.2 g/100 g), and guanosine 5′-monophosphate, uniquely facilitate their incorporation into diverse food formulations without requiring synthetic additives or extensive chemical modification [6].

From an environmental sustainability perspective, mushroom cultivation offers one of the lowest ecological footprints in protein production. Life cycle assessments demonstrate that mushroom protein production requires 85–90% less water and land use compared to conventional animal agriculture, with 80% lower greenhouse gas emissions, relative to beef and chicken production [10]. Mushroom cultivation systems can utilize agricultural waste streams including lignocellulosic byproducts, spent grain, and crop residues as growth substrates, supporting circular economy models and regenerative agricultural practices. These combined environmental advantages position mushrooms as strategically important components of sustainable food system transformation.

While previous reviews have addressed individual aspects of mushroom nutrition, specific bioactive compounds, or isolated product categories, no comprehensive and critically integrated analysis has simultaneously examined mushroom protein applications across the three most commercially significant product categories—meat analogs, functional snack products, and beverages—while systematically identifying cross-cutting research gaps. This review addresses that deficiency by establishing a techno-functional framework linking mushroom protein properties to processing technologies and product performance, critically evaluating the strength of evidence supporting health claims, assessing the environmental sustainability through the available life cycle data, and identifying priority research gaps in extraction methodology standardization, clinical validation, and techno-economic feasibility that currently limit large-scale commercialization. By integrating technological, nutritional, environmental, and economic dimensions within a single analytical framework, this review aims to advance the scientific and industrial foundation for mushroom-based proteins as viable mainstream alternatives to animal-derived protein ingredients.

This review focuses primarily on commercially cultivated edible mushroom species with established large-scale production systems—*Agaricus bisporus*, *Pleurotus ostreatus*, *P. eryngii*, *Lentinula edodes*, *Hericium erinaceus*, *Ganoderma lucidum*, *Cordyceps militaris*, and *Inonotus obliquus*—which collectively represent over 85% of global cultivated mushroom production. Wild species (e.g., *Astraeus odoratus*, *Russula* spp., *Termitomyces* spp.) are discussed solely to highlight exceptional nutritional attributes that may guide future domestication programs, not as currently viable commercial ingredients.

## 2. A Techno-Functional Framework for Innovative Applications

The successful development of mushroom-based food products including meat analogs, functional snacks, and functional beverages depends fundamentally on a thorough understanding of the nutritional composition, bioactive compound profile, and techno-functional behavior of edible mushroom species. The characterization presented in this section is not merely a descriptive background; it constitutes the scientific foundation that directly determines species selection, processing technology choices, formulation parameters, and achievable product quality outcomes in Section 3, Section 4 and Section 5. Parameters such as the water-holding capacity, gelation concentration, protein digestibility, β-glucan content, and thermal denaturation profiles translate directly into practical formulation decisions. This section therefore serves as an integrated reference framework connecting mushroom ingredient properties to the innovative product categories reviewed in subsequent sections.

### 2.1. Macronutrient Composition

Edible mushrooms possess a unique nutritional profile, making them highly suitable for incorporation into innovative food products. Their nutritional composition is summarized in Table 1, which presents the standard values derived from the USDA database and peer-reviewed research articles. Although nutritional content can vary depending on species and variety, general trends can be observed. Fresh mushrooms consist of 85–95% water; the dry matter contains approximately 19–35% protein, 32–61% carbohydrates (primarily dietary fiber), and 0.4–5.9% lipids, along with essential micronutrients [11]. The fatty acid profile of mushroom lipids is characterized by a favorable unsaturated-to-saturated ratio: saturated fatty acids (SFA) account for 15–30% of total lipids, monounsaturated fatty acids (MUFA) for 5–15%, and polyunsaturated fatty acids (PUFA) for 55–75%. Within the PUFA fraction, linoleic acid (ω-6, C18:2) predominates at 40–65% of total fatty acids, while α-linolenic acid (ω-3, C18:3) contributes 0.5–5%, reflecting an overall lipid profile that is more favorable than that of conventional meat products. Mushroom proteins are distinguished by their complete essential amino acid profile and superior digestibility compared to other plant-based sources [12]. The protein digestibility of edible mushrooms generally ranges from 60 to 80% (in vitro true digestibility), which substantially exceeds the values reported for many conventional plant protein sources. For comparison, the protein digestibility values for representative plant-based sources are approximately 63–78% for soybeans, 52–65% for chickpeas, 55–70% for lentils, 75–82% for wheat gluten, and 40–55% for raw cereals such as corn and rice [7,13]. The superior digestibility of mushrooms relative to most legumes and raw cereals is attributable to the absence of major antinutritional factors such as phytates and trypsin inhibitors, which are prominent in legume seed proteins and reduce protein bioavailability [14]. Interestingly, the amino acid composition includes all essential amino acids, with particularly high concentrations of lysine, leucine, and phenylalanine. Protein digestibility has been reported to range from 43.4% for Pleurotus sajorcaju to 80.50% for Agaricus macrosporus [15].

The carbohydrate fraction consists predominantly of complex polysaccharides, including β-glucans, chitin, and non-digestible fibers, which comprise over 50% of the dry matter [23]. These polysaccharides contribute significantly to both the health benefits and functional properties of mushroom-based food products. β-glucan concentrations vary among species, ranging from 16 to 35 g/100 g dry weight in cultivated varieties, while wild species such as Tricholoma portentosum demonstrate concentrations of up to 34.97 g/100 g dry weight [24]. This high β-glucan content distinguishes mushrooms from other plant-based protein sources and contributes to their functional food potential. The protein fraction of edible mushrooms also yields biologically active peptides upon hydrolysis, representing an additional dimension of protein value beyond basic nutrition, as described in Section 2.2.

### 2.2. Bioactive Peptides and Protein-Derived Health Benefits

Mushroom-derived bioactive peptides have emerged as a functionally important dimension of mushroom protein fractions, with diverse biological activities extending well beyond the conventional nutritional value. Enzymatic hydrolysis of mushroom proteins using food-grade proteases including Alcalase, pepsin, pancreatin, and papain generates peptide fractions with molecular weights typically ranging from 500 to 5000 Da, exhibiting antioxidant, antihypertensive, antimicrobial, and immunomodulatory activities that have important implications for functional food development [25].

Antioxidant peptides isolated from Agaricus bisporus, Pleurotus ostreatus, and Lentinula edodes protein hydrolysates demonstrate radical scavenging activity through multiple mechanisms including hydrogen donation, metal chelation, and free radical chain-breaking [26]. Peptides derived from Schizophyllum commune hydrolysates exhibited significant DPPH and ABTS radical scavenging activity, demonstrating that mushroom protein fractions can contribute meaningful antioxidant functionality beyond the phenolic compounds that are traditionally associated with mushroom antioxidant capacity. For instance, Goswami et al. (2021) [26] reported that Alcalase-derived *P. ostreatus* hydrolysates at 20% degree of hydrolysis achieved DPPH radical scavenging activity of 68.4% and ABTS scavenging of 72.1% in vitro. Similarly, peptides from *Schizophyllum commune* hydrolysates exhibited an IC_50_ of 0.87 mg/mL for DPPH scavenging in vitro. These activities are particularly relevant to protein bar and functional beverage applications, where the oxidative stability of bioactive components is a critical formulation challenge.

Antihypertensive peptides functioning as angiotensin-converting enzyme (ACE) inhibitors have been identified in protein hydrolysates of multiple commercially relevant mushroom species, suggesting potential applications in cardiovascular health-oriented functional foods [27]. The generation of ACE-inhibitory peptides through controlled enzymatic processing represents an important value-added opportunity in mushroom protein ingredient development, potentially enabling health claims beyond basic protein nutrition. Banjongsinsiri et al. (2016) reported that Alcalase hydrolysates of *L. edodes* achieved ACE inhibition with IC_50_ values of 0.43–1.12 mg/mL in vitro, which are comparable to the values reported for fish- and dairy-derived ACE-inhibitory peptides [28]. Notably, all reported antihypertensive activities are currently based on in vitro models; human clinical validation remains absent and represents a priority research gap. Antimicrobial peptides identified in mushroom protein hydrolysates offer additional functionality that is relevant to shelf-life extension in minimally processed mushroom-based products [28].

From a functional food application perspective, bioactive peptides offer particular advantages over intact proteins in beverage formulations where solubility and stability are critical and in fermented products where endogenous proteolytic activity during fermentation may enhance in situ peptide generation. However, research gaps remain: the stability of mushroom bioactive peptides during food processing (including thermal treatment, pH variation, and high-pressure processing) is incompletely characterized; clinical validation of in vitro bioactivities in human intervention studies is largely absent; and systematic peptide profiling across commercially important mushroom species using modern peptidomics approaches remains limited. Addressing these gaps represents a high-priority research direction for establishing mushroom proteins as multifunctional, health-promoting protein alternatives.

### 2.3. Micronutrient Profile

Mushrooms are exceptional sources of essential vitamins and minerals, contributing significantly to daily nutritional requirements. The vitamin profile is particularly noteworthy for B-complex vitamins, with the riboflavin (vitamin B_2_) content ranging from 1.8 to 5.1 mg/100 g, representing 129–364% of the RDI (1.4 mg/day for adults) [29]. The niacin (vitamin B3) content ranges from 31 to 65 mg/100 g, providing an impressive 194–406% of the RDI (16 mg/day). Folates (vitamin B9) are present at 0.30–0.64 mg/100 g, contributing 75–160% of the RDI (0.4 mg/day) [30]. These values demonstrate that even modest portions of mushrooms can substantially meet or exceed the daily requirements for these essential B vitamins. Uniquely among non-animal food sources, mushrooms provide vitamin D, particularly ergocalciferol (D2), which forms from ergosterol upon UV exposure. The vitamin D content ranges from <40 IU in commercially grown mushrooms to 1200 IU per 3.5-ounce serving in UV-exposed or wild varieties [31].

The mineral composition reveals potassium as the predominant macronutrient mineral (12.6–29.1 mg/g), followed by phosphorus (0.64–4.49 mg/g) and magnesium (0.90–4.54 mg/g) [32]. Among micronutrients, mushrooms contain significant concentrations of copper (0.1–0.5 mg/g dry weight), providing 1.1–5.6% of the RDI (0.9 mg/day) [33]. The iron content varies considerably among species (50.1–842 mg/kg), with varieties such as Boletus aereus, Cantharellus cibarius, and Pleurotus ostreatus showing particularly high concentrations. Zinc concentrations in mushrooms range from 26.7 to 185 mg/kg, making them a significant source of this essential mineral. Zinc is vital for immune function, protein synthesis, and DNA synthesis [34]. Table 1 presents the protein content and essential amino acid composition of major commercially cultivated edible mushroom species, providing a comparative overview of their nutritional value, relative to conventional meat sources and the recommended daily intake values.

The nutritional comparison between mushrooms and animal-based meat reveals several compelling advantages for mushrooms as functional foods and potential meat alternatives. While mushrooms provide lower protein content (1.9–3.3 g vs. 20–26 g per 100 g), they excel in several key micronutrients where meat falls short. Most notably, mushrooms deliver an exceptional copper content, providing 11–56% of the RDI compared to meat’s modest 9–17% contribution (Table 1). Mushrooms are uniquely positioned as one of the few non-animal sources of vitamin D, offering up to 800% of the RDI in UV-exposed varieties, while conventional meat contains virtually none (0–2 IU). The selenium content in mushrooms (2.6–26 µg) can provide up to 47% of the RDI, matching or exceeding meat’s selenium contribution [35]. Additionally, mushrooms offer significant advantages in dietary fiber (4–8% RDI vs. zero in meat), dramatically lower caloric density (22–37 kcal vs. 250–300 kcal), and complete absence of cholesterol [11]. The B-vitamin profile, particularly riboflavin (15–38% RDI) and pantothenic acid (30% RDI), demonstrates that mushrooms can effectively complement or partially substitute meat in nutritionally balanced diets, especially when considering their environmental sustainability and lower resource requirements for production [34].

### 2.4. Bioactive Compounds and Health Benefits

**Figure 1:**
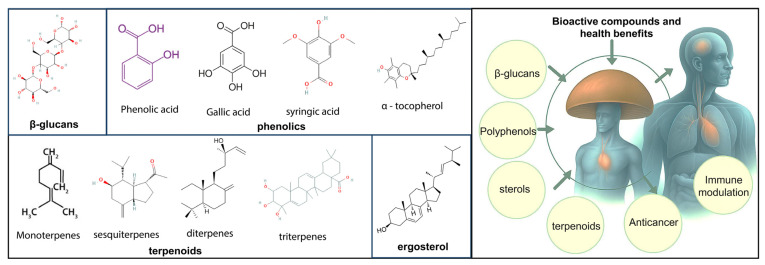
Chemical structure of some major bioactive compounds in mushrooms and its health benefits.

**Figure 2:**
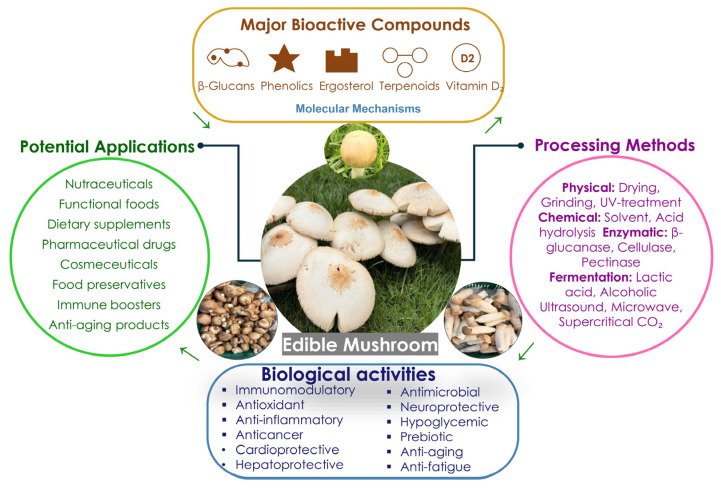
Comprehensive overview of bioactive compounds in edible mushrooms, their processing methods, biological activities, and potential applications.

Mushrooms are abundant in bioactive compounds, including β-glucans, phenolics, terpenoids, ergosterol, and a wide array of vitamins and minerals [36]. The structures of major bioactive compounds in mushrooms are presented in Figure 1, including their health benefits. These compounds exhibit a diverse array of biological activities, including prebiotic, immunomodulatory, antioxidant, hepatoprotective, anti-inflammatory, antihyperlipidemic, cytotoxic, anticancer, hypocholesterolemia, antidiabetic, antiallergic, antiviral, antibacterial, antiparasitic, antimicrobial, anti-fungal, free radical scavenging, cardioprotective, wound healing, and detoxifying effects [29]. It is important to note that the evidence base varies substantially across these activity categories. Immunomodulatory effects of β-glucans are supported by human clinical trials and antioxidant and anti-inflammatory activities are predominantly based on in vitro data, while anticancer, antidiabetic, antiviral, and cardioprotective effects are largely derived from in vitro cell line studies and animal models with limited human clinical evidence. Figure 2 illustrates the interconnected relationship between mushroom-derived compounds and their health benefits through various molecular mechanisms leading to nutraceutical and pharmaceutical applications.

Among these compounds, β-glucans are particularly important because of their immunomodulatory actions through contact with receptors such as Dectin-1 (C-type lectin domain family 7), which stimulate both innate and adaptive immune responses via epigenetic and metabolic reprogramming pathways [37]. Mushrooms’ β-glucans are characterized by β-1,3 backbones with β-1,6 branches, distinguishing them from the linear β-glucans found in cereals. This structural configuration confers diverse therapeutic effects including anticancer, antioxidant, cardioprotective, and antimicrobial activities, which are directly related to molecular weight, branching degree, and helical conformation [38].

Wild edible mushroom species were incorporated into this comparative analysis (Table 2) to provide a broader nutritional reference framework and to identify superior micronutrient profiles that may guide future targeted cultivation programs. Although hundreds of edible wild mushroom species are distributed globally, the species presented in Table 2 were systematically selected based on documented exceptional nutritional attributes, specifically elevated energy density, enhanced dietary fiber content, superior protein levels, and notable mineral concentrations, as reported in a peer-reviewed nutritional survey of wild species [39]. This targeted selection serves two purposes within the alternative protein context: first, it identifies wild species with protein content (up to 4.27 g/100 g fresh weight in Volvariella volvacea) that exceeds commercial varieties, providing a scientific rationale for domestication and controlled cultivation research; second, it highlights the substantial micronutrient advantages, particularly the exceptional calcium content in Astraeus species (185.6–193.4 mg/100 g) that may complement protein functionality in nutritionally fortified food product development [39]. As shown in Table 2, wild species such as *Astraeus odoratus* and *A. asiaticus* provide a significantly higher energy content (138–141 kcal/100 g) and exceptional dietary fiber levels (7.3–7.6 g/100 g), while *Russula c.f. kanadii* and *Volvariella volvacea* exhibit enhanced protein contents (4.19–4.27 g/100 g) compared to their commercial counterparts [39]. These wild varieties also demonstrate notable concentrations of essential minerals, with Astraeus species providing a particularly high calcium content (185.6–193.4 mg/100 g), positioning wild mushrooms as potentially valuable nutritional resources that warrant further investigation for sustainable cultivation and dietary integration [40].

The antioxidant capacity of mushrooms is mainly due to their various phenolic components, such as flavonoids and phenolic acids, which alleviate oxidative stress and lower the risk of chronic illnesses. Techniques like fermentation and enzymatic extraction improve the retention and bioavailability of these substances. Mushrooms are a unique non-animal source of vitamin D, notably ergocalciferol (D2), which is generated from ergosterol when exposed to ultraviolet light. A 75 g portion of UV-treated mushrooms can fulfill or surpass the recommended daily allowance of vitamin D. Furthermore, they are rich in B-complex vitamins and bioavailable B12 analogs, as well as ergothioneine, a powerful antioxidant with protective effects on mitochondria [41].

### 2.5. Techno-Functional Properties

**Figure 3:**
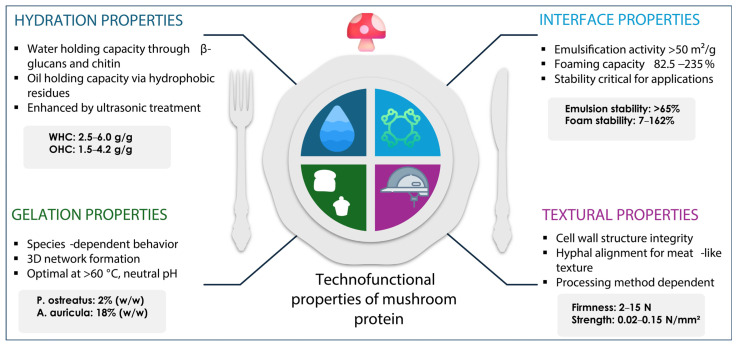
Techno-functional properties of mushroom proteins and their applications in food systems.

The successful integration of mushrooms into food formulations relies on their beneficial techno-functional properties, which can be categorized into several distinct functional characteristics that are essential for new food product development, as summarized in Table 3. Figure 3 shows the four key functional property categories: hydration properties, interface properties, foaming capacity, gelation properties, and with textural properties being critical for commercial applications.

#### 2.5.1. Water and Oil Holding Capacity

Water holding capacity (WHC) represents a critical parameter for maintaining texture and preventing moisture loss during processing. Mushroom-based ingredients demonstrate superior water retention capabilities, with values typically ranging from 2.5 to 6.0 g water per gram of dry matter. The high fiber content, particularly β-glucans and chitin, contributes significantly through hydrogen bonding and physical entrapment mechanisms [47].

Oil holding capacity (OHC) determines the ability to retain lipids and fat-soluble compounds, which are essential for texture development and flavor retention. Mushroom proteins exhibit oil holding capacities ranging from 1.5 to 4.2 g oil per gram protein, with variations depending on extraction methods and structural modifications. Ultrasonic and enzymatic treatments can enhance OHC by exposing hydrophobic amino acid residues [48].

#### 2.5.2. Emulsification and Foaming Properties

Emulsification properties are crucial for stabilizing oil–water interfaces in food formulations. Mushroom proteins demonstrate significant emulsification and foaming capabilities, with emulsification activity indices exceeding 50 m^2^/g and foaming capacities ranging from 82.5 to 235. Stability parameters prove more critical for food applications: emulsion stability indices surpass 65% at optimal pH 10 conditions, while foam stability varies widely from 7 to 162%, depending on extraction methods [49]. This comparison reveals that while initial capacity values demonstrate functional potential, stability parameters ultimately determine commercial viability in food systems. The amphiphilic nature of mushroom proteins enables effective interface stabilization, with spray-dried concentrates showing enhanced properties due to increased surface hydrophobicity [50]. For most food applications, emulsion and foam stability should be prioritized over the initial capacity, as these parameters directly impact the product shelf-life and texture maintenance.

#### 2.5.3. Gelation Properties

Gelation ability determines the capacity to form three-dimensional networks that entrap water and other components. Mushroom proteins demonstrate varying behaviors, depending on the species. For example, Pleurotus ostreatus protein requires a minimum concentration of only 2% (*w*/*w*) to form a gel, while Auricularia auricula protein needs a higher concentration of 18% (*w*/*w*) to achieve the same effect [51]. Heat treatment, pH adjustment, and ionic strength significantly influence the gelation behavior of protein, with optimal conditions typically occurring at temperatures above 60 °C and a neutral to slightly alkaline pH [52].

#### 2.5.4. Textural Properties

The texture of mushrooms is primarily determined by their cellular structure, with cell wall components (chitin, β-glucans, proteins) providing structural integrity. Fresh mushrooms exhibit firmness values typically ranging from 2 to 15 N when measured using texture profile analysis (TPA) with a cylindrical probe (typically 5–10 mm diameter) at compression speeds of 1–2 mm/s and 75–80% deformation [46]. Alternatively, firmness can be expressed as compressive strength ranging from 0.02 to 0.15 N/mm^2^, depending on species and maturity. *Pleurotus eryngii* demonstrates higher firmness (8–12 N; 0.08–0.12 N/mm^2^) compared to Agaricus bisporus (3–7 N; 0.03–0.07 N/mm^2^) [53]. The fibrous texture results from the alignment of hyphal structures, which contributes to the meat-like mouthfeel that is valuable in food applications. Processing methods significantly affect textural properties, with freeze-drying best preserving the original texture, while microwave drying often results in hardening and reduced rehydration.

### 2.6. Physicochemical Properties

The physicochemical properties of mushrooms strongly influence their processing behavior and functional performance in food applications. Understanding these properties is essential for optimizing their use in innovative products. Their specific contributions to product development are summarized in Table 4.

#### 2.6.1. pH and Acidity

The pH of fresh mushrooms typically ranges from 5.5 to 7.5, with most cultivated species falling in the range of 6.0–6.8. This slightly acidic to neutral pH range is optimal for nutrient availability and enzyme activity. Agaricus bisporus commonly exhibits pH values of 6.2–6.5, while Pleurotus species tend toward slightly higher (6.5–7.0) [57]. The pH significantly affects protein functionality, with optimal emulsification and foaming properties occurring at alkaline conditions (pH 10), while gelation is typically enhanced under neutral to mildly acidic conditions. Furthermore, post-harvest pH changes can serve as indicators of quality deterioration, with increasing acidity often correlating with senescence and microbial activity [58].

#### 2.6.2. Moisture Content and Water Activity

Fresh mushrooms contain 85–95% moisture, making water activity (a_w_) a critical parameter for processing and preservation. The water activity of fresh mushrooms typically ranges from 0.95 to 0.98, supporting the microbial growth, since their pH is not acidic and they require careful handling [59]. During dehydration processing, maintaining controlled moisture reduction is essential to preserve nutritional and functional properties. The optimal moisture content for dried mushroom products ranges from 8 to 12% to ensure stability, while maintaining rehydration capacity [60]. The equilibrium moisture content varies with the temperature and relative humidity, following typical food adsorption isotherms with values influenced by the hygroscopic nature of mushroom polysaccharides.

#### 2.6.3. Surface Properties

The surface hydrophobicity of mushroom proteins influences their functional properties, particularly their emulsification and foaming abilities. Studies using 1-anilino-8-naphthalenesulfonate as a fluorescent probe have revealed that fresh mushroom proteins typically exhibit moderate surface hydrophobicity, which can be enhanced through processing treatments such as ultrasonic treatment or enzymatic modification [61]. For instance, Pleurotus geesteranus proteins showed measurable surface hydrophobicity changes upon different processing conditions, directly correlating with improved emulsification properties [62]. The surface charge density varies with pH, showing typical protein behavior with isoelectric points generally occurring between pH 4–5 for most mushroom proteins [61]. These surface properties directly correlate with protein–protein and protein–water interactions, affecting the solubility and functionality in food systems.

#### 2.6.4. Thermal Properties

Thermal behavior of mushroom components is crucial for processing optimization. The thermal transition temperatures of mushroom proteins, typically between 60 and 85 °C, are indicative of their denaturation points, where the protein structure begins to unfold and lose its functional properties [63]. The thermal denaturation profile directly affects texture development and functional property retention during heat processing. In contrast, β-glucans and other polysaccharides demonstrate greater thermal stability, generally maintaining structure up to 120 °C, although extended heating may cause molecular weight reduction and property modification [64].

## 3. Mushroom-Based Meat Analogs

Meat analogs are products designed to replicate the sensory and nutritional characteristics of conventional meat while addressing sustainability and health concerns. Conventional materials for meat analog production primarily include soy protein isolates, wheat gluten, pea protein, and legume derivatives combined with binding agents like methylcellulose and carrageenan. These systems face limitations including allergenicity concerns, processing complexity, and consumer demand for minimally processed ingredients.

**Figure 4:**
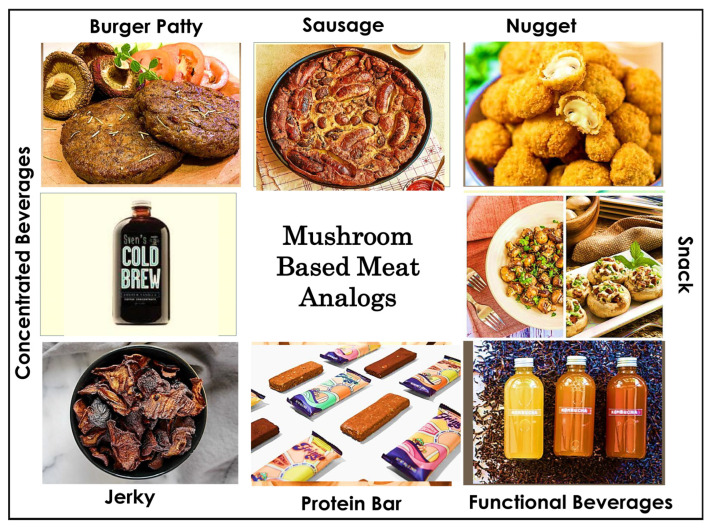
Diversity of mushroom-based meat analogs and innovative food products in current market applications, demonstrating the versatility of mushroom ingredients across product categories.

Mushroom-based products offer superior advantages, including natural umami compounds, fibrous meat-like textures, complete amino acid profiles, and bioactive compounds (β-glucans, ergothioneine). The integration of mushrooms into meat substitute systems represents a transformative approach to sustainable food innovation, offering significant health, environmental, and sensory benefits [65]. Some trending mushroom-based food products are shown in Figure 4 and global commercial companies and their products are presented in Table 5. It should be noted that several commercially available products listed in Table 5 are derived from mycelium—the vegetative, thread-like hyphal network of the fungal organism, rather than from mushroom fruiting bodies. Mycelium and mushroom fruiting bodies differ substantially in cellular structure, β-glucan linkage patterns, protein content, bioactive compound profiles, and techno-functional properties; nutritional and functional equivalence between mycelium-derived and fruiting body-derived ingredients cannot be assumed [66,67]. Mycelium-based products are included in Table 5, given their commercial significance within the broader fungal ingredient market, but this distinction is explicitly acknowledged. Future research and commercial product categorization should clearly differentiate between mycelium-derived and fruiting body-derived mushroom ingredients to ensure accurate nutritional claims and consumer transparency. The development of mushroom-based meat analogs has demonstrated considerable market potential, with the global plant-based meat market valued at USD 10.11 billion in 2022 and expected to grow at a compound annual growth rate (CAGR) of at least 15% by 2030 [68]. Recent developments in mushroom-based food products are shown in Table 6.

### 3.1. Burger Patty Development

Mushroom-based burger patties have demonstrated the ability to replace up to 50% of beef content while maintaining sensory acceptability. Their success depends on critical factors including species selection, moisture management, structural integrity optimization, and strategic binding system application.

#### 3.1.1. Species Selection and Processing Parameters

Pleurotus ostreatus (oyster mushrooms) has emerged as the preferred species for burger applications, due to its superior protein content (19–35% dry weight), balanced amino acid profile, and exceptional water retention capacity (4.5–6.0 g/g dry matter). Processing protocols typically involve controlled partial dehydration to reduce the moisture content to 75–80%, followed by mechanical processing for texture optimization [83]. Advanced processing techniques include cryogenic grinding at −196 °C to preserve the cellular integrity and ultrasonic treatment at 20 kHz for 15–20 min to enhance the protein functionality [84]. These methods improve the protein solubility and functional properties while maintaining nutritional value. However, species-specific optimization remains limited, with most studies focusing on *P. ostreatus* and Agaricus bisporus, indicating the need for systematic evaluation of other commercially viable species.

#### 3.1.2. Binding Systems and Formulation Optimization

Contemporary binding systems employ combinations of methylcellulose (0.5–1.0%), vital wheat gluten (2–4%), and legume-derived proteins (5–8%) to ensure patty cohesiveness and thermal stability [85]. Recent innovations include transglutaminase (0.1–0.3%) as a cross-linking agent, which significantly improves the texture and reduces cooking losses by up to 35% compared to conventional systems. The incorporation of alginate–pectin composite hydrogels (1.5–2.0%) has shown promise in enhancing moisture retention and providing a meat-like texture during consumption [86]. These binding systems work synergistically with mushroom proteins to create stable, cohesive products.

The available evidence suggests that while these binding systems show effectiveness, challenges persist in: (1) achieving clean-label formulations (many consumers avoid methylcellulose and modified ingredients), (2) ref. [87] maintaining texture stability across cooking methods (grilling, pan-frying, baking), and (3) standardizing the binding efficiency across different mushroom species and processing conditions. Furthermore, comparative studies evaluating different binding systems under identical conditions are lacking, limiting the optimization potential.

#### 3.1.3. Nutritional Profile and Sensory Characteristics

Mushroom-based burger formulations demonstrate superior nutritional characteristics compared to traditional beef patties, including 15–20% reduction in saturated fat content, a 25–30% increase in dietary fiber, and significant β-glucan incorporation (0.3–0.8 g per serving). The total caloric density is reduced by approximately 20–25%, while maintaining 12–18 g protein per 100 g serving with complete essential amino acid profiles. Sensory optimization leverages the natural presence of glutamic acid and 5′-nucleotides to develop umami flavor profiles, often eliminating the need for artificial enhancers. Properly formulated mushroom burger patties achieve consumer acceptance ratings of 7.2–8.1 on a 9-point hedonic scale [88].

However, variability in consumer acceptance (ranging 5.8–8.1 across studies) indicates the need for improved sensory optimization strategies. Challenges include: (1) earthy/mushroom off-flavors at higher incorporation levels (>40%), (2) ref. [87] texture differences from conventional beef (particularly juiciness and chewiness), (3) color stability during cooking and storage, and (4) limited shelf-life studies under retail conditions. Only 35% of the reviewed studies included comprehensive sensory panels with trained assessors and consumer testing.

### 3.2. Sausage Formulation and Processing Technology

**Figure 5:**
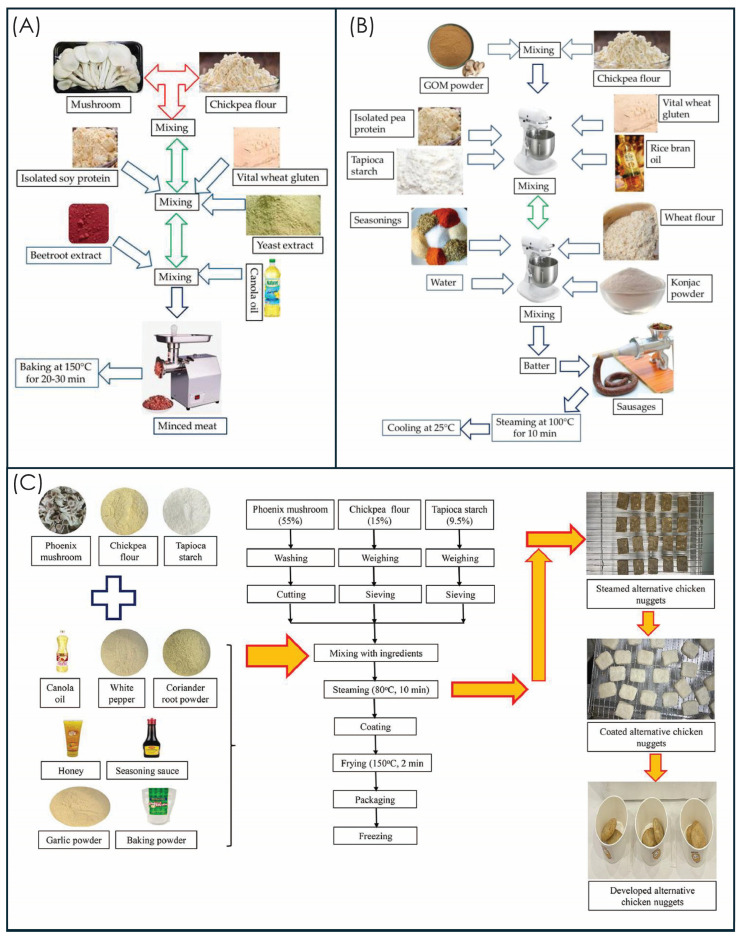
Comparative process flowcharts for mushroom-based meat analog preparation, demonstrating technological approaches for: (**A**) minced meat alternative [100], (**B**) sausage formulations [101], and (**C**) nugget production systems [72]. (GMO= Gray Oyster Mushroom).

Mushroom-based sausages offer extensive formulation flexibility for both fresh and processed varieties, with processing approaches emphasizing moisture control, fat substitution, and comprehensive flavor development. This segment represents one of the fastest-growing areas within meat alternatives, with annual growth rates of 15–19% in developed markets [73]. The complete procedure is shown in Figure 5B.

#### 3.2.1. Species Selection and Preparation Methods

*Agaricus bisporus* and *Pleurotus eryngii* demonstrate superior performance due to robust textural properties and neutral flavor profiles that facilitate seasoning integration. Processing involves precision cutting or mincing to achieve particle sizes of 2–5 mm, followed by controlled mixing with plant-based lipid systems (coconut oil, sunflower oil blends) and specialized seasoning formulations [89]. Pretreatment methods significantly influence the product quality, with blanching, steaming, and microwave treatments preferred over high hydrostatic pressure or UV treatments, which cause detrimental quality alterations [90]. The current literature indicates that processing parameter optimization remains species-specific and insufficiently standardized. Variations in particle size, pretreatment conditions, and lipid in-corporation methods across studies (coefficient of variation > 40%) limit reproducibility and industrial application. Additionally, the impact of different mushroom: lipid ratios on texture and mouthfeel requires systematic investigation.

#### 3.2.2. Advanced Binding and Emulsification Systems

Contemporary formulations employ sophisticated hydrocolloid systems, including sodium alginate (0.3–0.5%), carrageenan (0.5–0.8%), and konjac flour (1–1.5%), to create stable emulsion systems. Carrageenan at a 0.8% concentration has proven particularly effective, promoting water retention while reducing purge and cooking losses by more than 50% [91]. Additionally, high-intensity ultrasonic emulsification (20 kHz, 400 W, 5 min) creates stable lipid dispersions that enhance mouthfeel and cooking performance, representing an advancement in processing technology for plant-based sausages [92].

#### 3.2.3. Nutritional Enhancement and Quality Control

Mushroom-based sausages demonstrate significant nutritional advantages, including reduced sodium content (25–40% lower), elimination of saturated fats through plant-based lipid substitution, absence of nitrites and nitrates, and incorporation of prebiotic β-glucans (0.4–0.7 g per serving). Elevated dietary fiber content (8–12 g per 100 g) promotes digestive health and enhances satiety responses [93]. Optimal moisture content maintenance (60–65%) and pH control (5.8–6.2) are critical for product integrity and microbiological safety. Natural antimicrobial systems derived from mushroom extracts, containing ergothioneine and phenolic antioxidants, contribute to extended shelf life (7–10 days refrigerated, extendable to 21–28 days with modified atmosphere packaging) [94].

Recent developments include mushroom-based Northern Thai-style sausage, providing 34% (*w*/*w*) protein, 32% (*w*/*w*) dietary fiber (both on a dry weight basis), 44% of the sodium recommended daily intake [95], 10% of the calcium RDI, and 20% of the iron RDI. This product showed higher essential amino acid and polyphenol contents and higher DPPH activity than other mushrooms, and achieved better consumer acceptability and a comparable texture to traditional beef sausages [18]. Despite these advances, research gaps remain in: (1) long-term shelf-life stability studies (>30 days), (2) ref. [87] microbiological safety validation under temperature abuse conditions, (3) sensory stability during frozen storage, and (4) comprehensive nutritional bioavailability studies comparing fresh versus stored products.

### 3.3. Nugget Production and Advanced Processing Technologies

Mushroom-based nuggets serve as sophisticated alternatives to conventional poultry products, appealing to diverse consumer segments. The global plant-based nuggets market reached USD 1.3 billion in 2023, with projected compound annual growth rates of 14.7–18.9% through to 2030, driven by technological improvements and consumer demand [72].

#### 3.3.1. Protein Extraction and Functionalization

Mushroom protein extraction employs alkaline solubilization protocols (pH 10–11) at 50–60 °C for 2–4 h, followed by isoelectric precipitation at pH 4.5–5.0 to concentrate proteins and enhance functional properties [96]. Mushrooms with chickpea flour-based nuggets, particularly with a 55:15 ratio and tapioca starch, offer a promising soy-free alternative to commercial meat analogs with superior sensory quality and consumer acceptability [72]. The complete procedure to make the mushroom and legume-based nuggets is shown in Figure 5C. Further advanced techniques include enzyme-assisted extraction using alkaline proteases (0.1–0.3% *w*/*w*) and ultrasonic-assisted extraction (40 kHz, 200 W, 30 min) to improve yields and functionality [61]. The resulting protein concentrates achieve purity levels of 75–85% with enhanced solubility, emulsification capacity, and gelation properties. Deep eutectic solvent-based extraction methods demonstrate recovery rates of 21–22% compared to alkaline extraction (15%), while better preserving functional characteristics [97]. These concentrates serve as the foundation for texturized nugget products.

#### 3.3.2. Extrusion Technology and Texturization

High-moisture extrusion processing has emerged as the primary technology for making fibrous textures in mushroom-based nuggets. Optimal parameters include barrel temperatures of 130–150 °C, screw speeds of 100–160 rpm, and a moisture content of 55–65%. Mushroom-based texturized vegetable proteins (TVPs) (45–47% (*w*/*w*) protein) produced via single-screw extrusion showed a meat-like texture and acceptable sensory qualities, with King Oyster-based Sai-aua (traditional Northern Thailand-style sausages in which meat is replaced by King Oyster mushroom-based TVP) [98]. Twin-screw extruders with specialized die designs create striated, fibrous structures mimicking chicken breast’s texture. Typically, formulations with mushroom soy protein meat analogs (61–64% moisture) produced via high-moisture extrusion showed a protein content of 52.29–72.14%, improved intestinal digestibility, and the highest sensory acceptability, confirming that extrusion technology can improve the digestibility of mushroom-based meat analogs [99]. Despite technological advances, challenges remain in achieving consistent fiber formation across different mushroom species and scale-up from pilot to industrial production.

#### 3.3.3. Coating Systems and Cooking Technologies

Multi-stage coating processes employ aqueous batters containing modified starches (3–5%), plant-based proteins (2–3%), and hydrocolloids for enhanced adhesion, followed by textured breadcrumb applications. Cooking protocols utilize controlled oil frying at 175–180 °C for 3–4 min, with alternative methods including air-frying (200 °C, 12–15 min) reducing the oil content by 60–80% while maintaining the sensory characteristics [85].

## 4. Mushroom-Based Snack Products

The contemporary snack food industry has experienced unprecedented growth, with the global snack market valued at approximately USD 15.55 trillion in 2024 and projected to reach USD 19.7 trillion by 2032 [102], reflecting shifting consumer preferences toward convenient, nutritious, and functional foods. Once dominated by high-calorie, low-nutrient options, snacking is transforming as health-conscious consumers seek snacks that provide both taste and nutrition. Modern snacks now serve roles beyond satiation, offering energy between meals, nutrient supplementation, and portability. This paradigm shift includes ready-to-eat products, protein bars, functional snacks, and specialized formulations designed for specific dietary needs while ensuring palatability and shelf life. Mushrooms are redefining the snack food sector with their nutrient density, umami flavor, and sustainable production characteristics. They meet the consumer demand for higher protein, dietary fiber, bioactive compounds, and plant-based options. Their inherent umami enhances flavor while lowering sodium requirements, and bioactive like β-glucans, ergothioneine, and phenolics offer functional health benefits. Mushrooms adapt well to diverse formats, from dehydrated crisps to protein-enriched extrusions and fermented snacks [103].

The commercial mushroom-based snack sector encompasses chips, jerkies, functional snacks, and beverages formulated from both culinary and medicinal species. Premium mushroom chips and crisps, such as those from other foods, feature shiitake (*Lentinula edodes*), oyster (*Pleurotus ostreatus*), and king trumpet (*Pleurotus eryngii*) mushrooms, employing advanced dehydration to preserve nutrients, while mixed chip assortments including shiitake, button (*Agaricus bisporus*), and oyster varieties are available through platforms like Amazon [104,105]. Mushroom jerkies leverage shiitake’s robust umami and firm texture to deliver vegan, fiber-rich, allergen-friendly products with desirable chewiness. Functional snack lines integrate mushrooms into jerkies, snack bars, chips, and ready-to-drink beverages, often utilizing functional species including Reishi (*Ganoderma lucidum*), lion’s mane (*Hericium erinaceus*), and cordyceps (*Cordyceps militaris*) for their bioactive compound content. Species-specific attributes guide product development: shiitake is valued for umami intensity and structural integrity; oyster mushrooms offer mild flavor and optimal dehydration properties; button mushrooms provide broad consumer acceptability; and king trumpet mushrooms contribute a substantial texture that withstands processing.

### 4.1. Ready-to-Eat Snack Development

#### 4.1.1. Extrusion-Based Mushroom Snacks

Extrusion processing has emerged as a primary technology for mushroom-based savory snack production, utilizing controlled thermal and mechanical energy to create expanded, shelf-stable products. Optimal processing parameters for mushroom–corn extrudates include 15% (*w*/*w*) Pleurotus ostreatus powder incorporation, 13.5% (*w*/*w*) feed moisture content, 425 rpm screw speed, and 130 °C barrel temperature, producing snacks with expansion ratios of 4.2–5.8 [106].

Advanced twin-screw extrusion systems operating at specific energy inputs of 180–220 Wh/kg create optimal protein structure modifications while preserving bioactive compounds. Mushroom powder incorporation at 10–20% concentrations result in products containing 18–25% protein, 8–12% dietary fiber, and significant β-glucan concentrations (0.8–1.2 g/100 g) [107]. However, challenges remain in maintaining consistent expansion ratios and textures across different mushroom species and moisture levels. A critical evaluation indicates that expansion ratio variability (coefficient of variation 15–28%) across studies limits process standardization, while effects of different mushroom species on expansion behavior remain insufficiently characterized.

#### 4.1.2. Advanced Drying Technologies

Contemporary drying methodologies have revolutionized mushroom snack production. Freeze-drying emerges as the superior technique for preserving nutritional and functional properties, retaining 86% of ascorbic acid content and maximum antioxidant activity compared to conventional methods. The process, conducted at −40 °C under 0.1 mbar vacuum, maintains cellular structure while achieving moisture contents below 5%, ensuring 12–18 month shelf life [108]. Vacuum microwave drying at 400 W power and 75 kPa pressure represents an economically viable alternative, achieving 78–82% antioxidant preservation with significantly reduced processing times (4–6 h versus 24–48 h for freeze-drying) [109].

#### 4.1.3. Fermented Mushroom Snacks

Probiotic mushroom snacks represent an innovative category combining controlled fermentation with functional ingredients. Fermentation protocols utilizing *Lactobacillus plantarum* and *L. acidophilus* at 37 °C for 48–72 h enhance flavor while incorporating beneficial microbial cultures (10^8^–10^9^ CFU/g). During fermentation, pH values decrease from initial levels of 6.2–6.5 to final values of 3.8–4.2, creating acidic conditions that inhibit pathogenic microorganisms while promoting desirable flavor development and texture modification. Mushroom-derived β-glucans promote probiotic proliferation, creating synergistic health benefits [110]. Despite promising results, several challenges persist: (1) probiotic viability during storage decreases 1–2 log cycles over 30 days under refrigeration, (2) ref. [87] sensory acceptability varies widely (acceptance scores 5.8–7.9/9.0) depending on fermentation intensity and mushroom species, (3) standardization of fermentation parameters across different mushroom matrices remains incomplete, and (4) shelf-life stability of probiotic counts under different packaging and storage conditions requires systematic investigation. Only 12% of reviewed fermented mushroom snack studies included comprehensive shelf-life evaluations beyond 30 days [111].

### 4.2. Protein Bar Innovation and Functional Enhancement

The integration of mushrooms into protein bar formulations has gained momentum within functional foods and sports nutrition markets. The global protein bar market, valued at USD 14.18 billion in 2023 and projected to reach USD 20.87 billion by 2030, increasingly features mushroom-based ingredients [112].

#### 4.2.1. Advanced Protein Extraction and Processing

Contemporary extraction employs multi-stage processes combining enzymatic hydrolysis and alkaline solubilization to achieve protein concentrates with 75–85% purity and complete amino acid profiles. Alkaline extraction at pH 10–11, followed by isoelectric precipitation, yields fractions with enhanced functional properties including improved solubility (>90% at pH 7) and water-holding capacity (5.8–7.2 g/g) [113]. Enzymatic pre-treatment using alkaline proteases’ commercial preparation (AlcalaseTM, 0.2% *w*/*w*) at 55 °C for 3 h increases the extraction efficiency by 35–42% while maintaining bioactive peptide integrity [114]. However, scalability challenges and variations in extraction efficiency across mushroom species (extraction yields ranging 12–28%, depending on species and methods) indicate a need for standardized protocols.

#### 4.2.2. Commercial Product Development and Critical Evaluation

Analysis of commercial products reveals that leading formulations incorporate standardized functional mushroom extracts containing Reishi (*Ganoderma lucidum*), lion’s mane (*Hericium erinaceus*), cordyceps (*Cordyceps militaris*), and chaga (*Inonotus obliquus*) at therapeutic concentrations of 1000–1500 mg per serving. Clinical trials demonstrate 12–18% improvement in memory recall and attention span following 6-week supplementation periods [87]. Optimized formulations contain 15–18 g high-quality protein, maintain caloric density below 200 kcal per 40 g serving, and limit added sugars to <5 g while incorporating natural sweetening systems [115]. Critical analysis reveals that while these products show promise, several challenges persist: (1) inconsistent bioactive content standardization across manufacturers (inter-product variations up to 40% for same claimed species/dose), (2) ref. [87] limited long-term clinical validation of health claims (only 23% of commercial products have supporting human intervention trials >8 weeks duration), (3) taste and texture acceptability concerns (consumer acceptance scores averaging 6.8 ± 1.2/9.0, lower than conventional protein bars at 7.6 ± 0.8/9.0), and (4) premium pricing (typically 2–3× conventional protein bars), limiting market accessibility. Quality assurance protocols achieving 20–25% protein content by weight, controlled moisture levels (8–12%), and comprehensive microbiological testing remain essential but inconsistently implemented across the industry. Furthermore, bioavailability studies comparing mushroom protein versus conventional sources (whey, soy, pea) are lacking in 89% of the reviewed product development literature.

### 4.3. Baked Snack Applications

Mushroom flour incorporation at 5–15% (*w*/*w*) in baked applications enhances nutritional profiles through protein, fiber, and antioxidant fortification. Incorporating mushroom flour at 5–10% in cracker formulations increases the protein content by 25–40%, dietary fiber by 35–50%, and antioxidant activity by 60–80% compared to conventional products. Pleurotus flour incorporation at 7% (*w*/*w*) demonstrates an optimal balance between nutritional enhancement and sensory acceptability, achieving consumer preference scores of 7.8–8.2 on 9-point hedonic scales [116]. Advanced processing includes controlled fermentation using sourdough cultures to reduce earthy flavors while enhancing bioavailability. Mushroom-enriched products demonstrate enhanced antimicrobial and antioxidant properties due to naturally occurring phenolic compounds and ergothioneine, contributing to extended shelf life through the inhibition of lipid oxidation and microbial growth [117].

Research gaps persist in flavor-masking mechanisms and optimizing processing conditions to balance sensory quality with nutritional benefits. Key challenges include: variable earthy flavor detection thresholds (3–12% mushroom flour, depending on species and consumer sensitivity); texture changes at higher incorporation levels (hardness +20–45%, fracturability 15–30%); baking-induced browning (+25–60% by species); moisture migration affecting crispness during storage; and a limited understanding of mushroom flour–gluten interactions in dough rheology. Consumer education on earthy flavor profiles and health benefits also remains critical, as blind taste tests show 30–40% lower acceptance versus informed tasting conditions.

## 5. Functional Mushroom Beverages

Functional beverages constitute a rapidly expanding sector within the global beverage industry. These products incorporate bioactive ingredients such as botanical extracts, probiotics, adaptogens, and specialized compounds to deliver physiological benefits beyond basic nutrition, including immune function enhancement, cognitive support, and metabolic optimization [118]. Traditional formulations utilize scientifically validated ingredients including Camellia sinensis extracts, Panax ginseng, Curcuma longa, and probiotic cultures to create diverse product categories ranging from energy drinks to fortified waters.

The integration of mushrooms into functional beverages represents an innovative evolution leveraging unique fungal bioactive properties for enhanced therapeutic potential. The mushroom beverage market, estimated at USD 3.70 billion in 2023 with 6.7% CAGR through 2030, demonstrates significant commercial adoption driven by mushrooms’ distinctive bioactive profile, including β-glucans, triterpenes, ergothioneine, and immunomodulatory polysaccharides [119]. Species such as *Ganoderma lucidum*, *Hericium erinaceus*, *Cordyceps militaris*, and *Inonotus obliquus* provide synergistic umami flavor enhancement while delivering neuroprotective and adaptogenic effects. Commercial applications include Four Sigmatic’s organic coffee blends with lion’s mane and chaga, Om Mushroom Superfood’s matcha formulations, and Laird Superfood’s multi-mushroom functional blends, demonstrating successful mainstream integration of fungal bioactives in consumer beverage formats [120].

### 5.1. Non-Alcoholic Fermented Mushroom Beverages

Non-alcoholic fermented mushroom beverages have emerged as a sophisticated category within functional drinks, utilizing kombucha-style fermentation enhanced with mushroom substrates. The global kombucha market reached USD 2.59 billion in 2021, with a projected CAGR of 15.7% through to 2030 [121].

#### 5.1.1. Fermentation Technology and Process Optimization

Contemporary mushroom kombucha production employs SCOBY-based systems (Symbiotic Cultures of Bacteria and Yeasts) augmented with specific mushroom species including Ganoderma lucidum, Lentinus edodes, and Coriolus versi-color. Optimized parameters include temperature control at 22–26 °C, pH monitoring from the initial 4.5 to final 3.2–3.5, and fermentation periods of 7–14 days. The microbial community includes acetic acid bacteria (*Komagataeibacter*, *Acetobacter*, *Gluconobacter*), lactic acid bacteria (*Lactobacillus species*), and yeasts (*Saccharomyces*, *Brettanomyces*, *Zygo-saccharomyces*) that transform mushroom-derived carbohydrates into organic acids, enhancing preservation and developing complex flavor profiles [114].

However, several challenges limit widespread commercialization: batch-to-batch variability in microbial composition (20–45% relative abundance variation among key species); inconsistent bioactive content across batches (β-glucan concentrations, ranging 2.1–15.2 mg/100 mL); limited understanding of mushroom polysaccharide SCOBY interactions; sensory concerns (vinegar-like acidity perceived negatively by 35–48% of untrained consumers); and regulatory uncertainties around health claims and alcohol content. The standardization of starter culture composition and fermentation conditions remains a critical research priority, yet has been addressed in only 27% of reviewed studies.

#### 5.1.2. Bioactive Compound Enhancement

Fermentation significantly enhances β-glucan bioavailability, increasing concentrations from 2.1 to 3.8 mg/100 mL in unfermented preparations to 8.5–15.2 mg/100 mL in completed beverages. Standardized quality parameters include maintaining viable probiotic counts of 10^8^–10^9^ CFU/mL, alcohol content below 0.5% (*v/v*), and pH values of 3.5–4.0. HPLC-MS/MS analysis confirms the presence and stability of key bioactive compounds, including triterpenes, ergosterol, and immunomodulatory polysaccharides throughout 30–60 days of refrigerated shelf life [122]. Although bioactive enhancement is established, gaps remain: unclear β-glucan modifications during fermentation; limited study of metabolite–bioactive synergy (~15%); scarce human bioavailability data; inadequate storage stability assessment; and absence of cost–benefit analyses in 91% of studies [123].

### 5.2. Concentrated Mushroom Extract Beverages

Concentrated extract beverages represent the premium functional beverage segment, targeting consumers seeking therapeutic and adaptogenic benefits from standardized, high-potency formulations.

#### 5.2.1. Advanced Extraction Technologies

Contemporary extraction employs dual-phase systems combining hot water extraction (90–95 °C, 2–4 h) and ethanol extraction (70% *v*/*v*, 24 h) to maximize the recovery of both water-soluble polysaccharides and alcohol-soluble triterpenes. Ultrasonic-assisted extraction (40 kHz, 500 W, 45 min) increases the polysaccharide yields by 28–35% while maintaining the bioactivity. Supercritical CO_2_ extraction at 350 bar pressure and 60 °C provides highly pure extracts that are free from solvent residues, which are particularly valuable for organic certification and clean-label compliance [124]. Key challenges include low and variable extraction efficiency, loss of thermolabile compounds, solvent residue issues, high supercritical CO_2_ costs, limited method comparisons, and poor scale-up optimization that is only systematically addressed in 19% of studies [125].

#### 5.2.2. Standardization and Quality Assurance

Commercial extract beverages are standardized to contain 20–30 mg/100 mL total β-glucans with specific β-1,3 and β-1,6 linkage ratios verified through enzymatic digestion and Congo red dye binding assays. Triterpene content is standardized at 5–15 mg/100 mL depending on the species, with ganoderic acids serving as marker compounds for Ganoderma-based products [125]. Flavor-masking systems utilize natural agents, including stevia leaf extract, monk fruit concentrate, and organic fruit flavors combined with complementary botanicals. Microencapsulation using modified starch matrices (5–8% *w*/*v*) and spray-drying reduces the perceived bitterness by 40–60% while maintaining the bioactivity. Quality control gaps include inconsistent β-glucan quantification, limited validated triterpene methods, non-standardized bioactivity assays, inadequate shelf-life stability data, weak links between analytical markers and clinical efficacy (only 8% validated), and poor authentication against adulteration in 76% of products [124].

### 5.3. Alcoholic Mushroom Beverages

Alcoholic mushroom beverages represent an innovative convergence of traditional brewing with functional mushroom integration, appealing to consumers seeking artisanal, functional alcoholic products. Direct fermentation of mushroom biomass using specialized yeast strains achieves alcohol concentrations of 8–12% (alcohol by volume, ABV), while maintaining bioactive compounds’ integrity. Hybrid production systems combine mushroom extract integration with conventional brewing substrates to create beverages with 4–8% ABV, characterized by complex umami-rich flavor profiles and reduced artificial additive requirements [126]. This category remains constrained by a limited evidence base, regulatory barriers to functional claims, consumer perception conflicts, bioactive instability during alcoholic fermentation, sensory challenges, and unclear market positioning. Comprehensive sensory, stability, and consumer acceptance studies are scarce (13%), and the potential health risks of combining alcohol with bioactive compounds remain insufficiently evaluated.

## 6. Processing Technologies and Quality Considerations

The successful commercialization of mushroom-derived functional products necessitates the implementation of advanced processing technologies that optimize bioactive compound preservation while ensuring product safety, stability, and consumer acceptability. Contemporary food processing has evolved beyond traditional thermal methods to encompass innovative non-thermal technologies, including high-pressure processing (HPP), pulsed electric fields (PEF), ultrasonic-assisted extraction, and precision fermentation systems that selectively target pathogenic microorganisms, while maintaining the structural integrity of thermolabile bioactive compounds such as β-glucans, triterpenes, and ergothioneine [127]. These novel processing approaches address the fundamental challenge of mushroom-based product development: maximizing functional compound bioavailability and stability while achieving acceptable organoleptic properties, extended shelf-life, and comprehensive microbiological safety through integrated quality assurance frameworks that encompass analytical validation, contaminant monitoring, and accelerated stability testing protocols [128].

The successful development of mushroom-derived functional products requires precise control of processing parameters and rigorous quality assurance protocols to preserve bioactivity and organoleptic quality, while ensuring microbiological safety and chemical stability. Various novel techniques have been employed to create innovative mushroom-based foods for different applications and benefits, as summarized in Table 7.

### 6.1. Thermal Processing Optimization

Conventional thermal processing requires careful optimization to preserve essential bioactive compounds. Steam blanching at 85–90 °C for 2–3 min effectively inactivates polyphenol oxidase and peroxidase enzymes while maintaining the structural integrity of β-1,3 and β-1,6 glucan linkages. Extended thermal exposure above 100 °C for periods exceeding 15 min compromises thermolabile nutrients including ergothioneine (33–59% losses during thermal processing) [135], ascorbic acid (degradation accelerated >90 °C), and indole compounds, which are susceptible to thermal decomposition [47]. Critical challenges include species-specific thermal sensitivity, poorly understood heat-induced β-glucan modifications, limited multi-objective process optimization, scale-up heat transfer issues, and inadequate kinetic modeling; only 24% of studies apply systematic optimization methods.

### 6.2. Non-Thermal Processing Technologies

High-pressure processing (HPP) at 400–600 MPa for 3–5 min represents a superior non-thermal preservation method, destroying pathogenic microorganisms through cell membrane disruption while preserving nutritional and functional characteristics. This technology extends shelf life to 21–28 days under refrigeration, making it particularly suitable for functional beverages and minimally processed applications [136]. Pulsed electric field (PEF) processing at 25–35 kV/cm with treatment times of 100–500 μs demonstrates effectiveness in microbial inactivation while maintaining enzyme activity and bioactive compound stability [137].

### 6.3. Ultrasonic-Assisted Processing

Ultrasonic treatment at frequencies of 20–40 kHz with power densities of 0.5–2.0 W/mL disrupts cellular matrices through cavitation effects, increasing β-glucan release by 35–45% while maintaining the molecular weight and branching complexity that are essential for biological activity. Ultrasonic-assisted extraction optimized via an RSM (Response Surface Methodology) effectively enhanced the yield of A. bisporus polysaccharides (ABPS), yielding a Glc-based β-glycosidic polysaccharide with notable antioxidant activity [138]. These findings highlight ABPS’s potential as a natural antioxidant in functional food applications. Ultrasonic processing faces key limitations, including scale-up challenges due to reduced cavitation in large volumes, equipment fouling by mushroom particles, possible bioactive degradation at high intensities, high energy demand (0.8–2.5 kWh kg^−1^), limited penetration in viscous systems (<20 cm), and batch variability in extraction efficiency (12–28% CV). Moreover, multi-objective optimization studies account for only 21% of the literature, and industrial-scale validation (>100 L) for just 8% of reported methods [138].

### 6.4. Microencapsulation and Stabilization

Advanced microencapsulation technologies, particularly spray-drying using ultrasonic nozzles, protect mushroom-derived bioactive compounds from oxidation, preserving their antioxidant, immunomodulatory, anti-inflammatory, and antimicrobial activities. Encapsulation matrices including maltodextrin (Dextrose Equivalent (DE): 10–15), modified starches, and whey protein isolates provide particle uniformity and bioactivity preservation [139]. Fluid bed coating using enteric polymers enables the targeted release of bioactive compounds in specific gastro-intestinal regions, enhancing bioavailability and therapeutic efficacy [140].

### 6.5. Comprehensive Quality Assurance Framework

Ensuring the quality and safety of mushroom-based food products requires a systematic approach to bioactive analysis, contaminant control, and stability assessment. β-glucan, a key functional compound, is quantified using enzymatic assays and Congo red dye binding, with the latter offering a higher correlation with biological activity. Advanced techniques such as NMR and HPLC-MS/MS support the detailed profiling of triterpenes, ergosterol, and bioactive peptides. Due to mushrooms’ tendency to accumulate heavy metals, routine testing for lead, cadmium, mercury, and arsenic is essential to meet food safety standards. Mycotoxins (e.g., aflatoxins, ochratoxin A) are detected via ELISA and LC-MS/MS, while microbial safety is assessed through pathogen panels for Salmonella, *E. coli*, and Listeria [141].

Stability and shelf-life are evaluated through accelerated (40 °C, 75% RH) and real-time studies, focusing on bioactive degradation, microbial stability, and sensory quality retention. Packaging solutions use oxygen-barrier materials (<0.01 cc/100 in^2^/day) with desiccants or scavengers, while nitrogen flushing in modified atmosphere packaging extends shelf life and preserves functionality [142].

Critical gaps in quality assurance include: lack of harmonized international standards for β-glucan quantification; limited validated methods for simultaneous multi-mycotoxin detection in mushroom matrices; insufficient correlation between accelerated and real-time stability data (predictive models available for only 18% of product types); incomplete understanding of packaging material–bioactive interactions; limited real-time monitoring in production; and absence of standardized bioactivity testing protocols throughout shelf life, with most studies measuring the chemical content, rather than the biological function. Additionally, traceability systems linking cultivation conditions to the final product quality remain underdeveloped, hindering the root cause analysis of quality deviations.

## 7. Market Trends and Consumer Acceptance

### 7.1. Market Growth and Commercial Opportunities

The worldwide functional mushroom market has rapidly expanded; it was estimated at USD 31.09 billion in 2024 and is anticipated to reach USD 62.18 billion by 2032, with a CAGR of 9.14% by [143]. The mushroom supplement sector in North America attained USD 541.77 million in 2023, with a projected CAGR of 15.0% until 2030 [144]. Growth is primarily propelled by heightened consumer health consciousness and the growing scientific endorsement of bioactive constituents, especially β-glucans.

Meta analyses of more than 100 scientific research studies have validated the immunomodulatory effectiveness of mushroom-derived β-glucans, demonstrating a 30–50% improvement in immunological responses [145]. The COVID 19 pandemic intensified the interest in products that enhance natural immunity. Product innovation has expanded beyond supplements to include functional coffees, chocolates, and cosmetic formulations, signifying substantial economic potential within the lifestyle and wellness sectors [146].

### 7.2. Consumer Acceptance and Sensory Optimization

Consumer acceptance is significantly influenced by sensory characteristics, with umami flavor enhancement serving as a key acceptance driver. Natural glutamate content (2.8–4.2 g/100 g dry weight) provides authentic umami characteristics while enabling sodium reduction strategies [147]. Generational analysis reveals significant adoption differences, with Gen Z consumers showing 37% engagement with mushroom-infused foods compared to 22% among Baby Boomers. This demographic shift reflects changing attitudes toward functional foods and willingness to try innovative ingredients. Consumer research indicates that products containing up to 15% mushroom powder maintain high acceptability scores (>7.0/9.0) when properly formulated with complementary flavoring systems. Key acceptability factors include texture optimization, earthy flavor masking, and clear communication of health benefits.

Sustainability considerations significantly influence consumer choice, with 68% preferring plant-based supplements over synthetic alternatives, according to the Global Wellness Institute 2023. Mushroom cultivation’s minimal environmental footprint, requiring 90% less water and generating 80% fewer greenhouse gas emissions compared to animal protein production, aligns with consumer preferences. Life cycle assessments demonstrate that mushroom protein production requires 85% less land use and 75% less energy input compared to conventional animal agriculture, supporting the marketing claims of sustainable nutrition.

### 7.3. Regulatory Framework and Health Claims

Regulatory frameworks vary significantly across regions but are progressively evolving to accommodate mushroom-based functional foods. In the United States, specific mushroom-derived compounds including β-glucans are generally recognized as safe (GRAS), while health claims require substantial clinical validation. Toxicological evaluations establish no observed adverse effect levels (NOAEL) up to 2000 mg/kg/day for mushroom-derived β-glucans, supporting safe consumption at therapeutic dosages [148]. The Food and Drug Administration (FDA)’s guidance allows structure–function claims when supported by appropriate scientific evidence.

The European Food Safety Authority [149] has established precedents for β-glucan health claims for cereal sources, though mushroom-derived β-glucans await specific authorization despite their demonstrated equivalent biological activity [149]. Current research into bile acid-binding capacity and immunomodulatory properties may facilitate future regulatory approval. International markets including Japan, Canada, and Australia have established comprehensive regulatory frameworks supporting functional food innovation. Japan’s Foods for Specified Health Uses (FOSHU) system and Canada’s Natural Health Products Regulations provide clear pathways for mushroom-based product approval.

Meta-analyses of over 150 clinical studies confirm the immunomodulatory effects of mushroom-derived β-glucans, showing 30–50% improved immune responses and reduced incidence of upper respiratory tract infections. Regulatory recognition varies: in Japan, specific mushroom β-glucan products have FOSHU approval for immune and gut health; in the EU, EFSA has approved cholesterol-lowering claims for oat/barley β-glucans, with mushroom-derived forms still being under review; in the US, they are sold as dietary supplements with structure/function claims but no FDA-approved health claims [150]. Ongoing clinical trials investigating cognitive enhancement, metabolic health, and cancer adjuvant therapy applications may substantially expand the approved health claims, potentially transforming the market opportunities for mushroom-based functional foods [151].

## 8. Future Perspectives and Research Directions for Mushroom-Based Food Products

Technological advancements are rapidly transforming mushroom-based food production, offering scalable, sustainable, and innovative solutions. Precision fermentation using engineered *Saccharomyces cerevisiae* and *E. coli* enables high yield synthesis of bioactive compounds, outperforming traditional cultivation methods by 10–15 times [152]. Synthetic biology platforms, such as CRISPR-Cas9-enabled *Aspergillus oryzae*, have facilitated targeted enhancements in nutritional and sensory profiles, including increased production of ergothioneine and heme for meat analogs. CRISPR-Cas9 applications in edible mushrooms like *Pleurotus eryngii*, *Cordyceps militaris*, and *Agaricus bisporus* have improved the yield, metabolite biosynthesis, and stress resistance [22].

Advancements in green extraction techniques, including supercritical CO_2_ (300–400 bar, 40–60 °C), microwave-assisted, and enzyme-assisted methods, significantly enhance the extraction efficiency (up to 95%) while reducing the environmental impact and processing time [153]. These approaches ensure the recovery of high-purity, functional compounds with minimal degradation.

Artificial intelligence and machine learning are revolutionizing mushroom cultivation through real-time data-driven optimization. Predictive models integrating genomics, metabolomics, and environmental data have achieved up to 35% increases in bioactive yields and improved production efficiency [154]. Further personalized nutrition is emerging as a critical application of mushroom bioactive compounds. Nutrigenomic studies reveal that genetic polymorphisms in receptor enzymes influence individual responses to β-glucans and triterpenes, informing precision dosing strategies [155]. The gut microbiome further modulates these effects, with specific microbial profiles affecting compound bioavailability and efficacy [156].

Digital health tools, including wearable biosensors and AI-driven mobile applications, support the real-time monitoring and optimization of mushroom-based functional food intake, demonstrating up to 60% improvement in health outcomes compared to conventional dietary recommendations. From a sustainability perspective, mushroom cultivation presents one of the lowest environmental footprints in protein and functional ingredient production, with carbon emissions [157]. Life cycle assessments show that mushroom farming uses up to 90% less water and land, and emits 80% fewer greenhouse gases compared to animal agriculture. Circular economy models utilize agricultural residues as substrates, while spent mushroom substrate (SMS) enhances soil health and fertility. Integrated industrial systems incorporating waste heat and CO_2_ further reduce the overall environmental impact. Moreover, mushroom-based systems support regenerative agriculture through mycorrhizal networks that improve soil structure, nutrient cycling, and biodiversity. Co-cultivation with cover crops has been shown to enhance soil organic matter and reduce erosion, advancing sustainable agriculture practices [158].

## 9. Conclusions

Edible mushrooms represent viable sustainable protein alternatives, delivering 19–35% protein (dry weight) with complete essential amino acid profiles and digestibility rates of 60–80%. This review provides the first integrated analysis across meat analogs, functional snacks, and beverages within a unified techno-functional framework, with a new dedicated treatment of mushroom-derived bioactive peptides (ACE-inhibitory, antioxidant, antimicrobial) as a distinct protein value dimension. β-glucans (16–35 g/100 g), advanced processing technologies including high-moisture extrusion and microencapsulation, and environmental advantages (85–90% less water and land use than animal agriculture) further strengthen their commercial case. However, critical gaps persist: extraction efficiency varies 3-fold across studies; only 23% of commercial products are supported by clinical trials; techno-economic analyses are absent in 78–82% of studies; and mycelium versus fruiting body ingredient distinctions remain unstandardized across commercial and regulatory frameworks. Future priorities include large-scale clinical validation, harmonized quality standards, systematic peptidomics profiling, and comprehensive techno-economic analyses. Mushroom-based proteins possess the scientific foundation and market potential to become mainstream sustainable protein alternatives with continued interdisciplinary investment.

## Figures and Tables

**Table 1:** General nutritional facts of edible mushrooms per 100 g (wet basis) with RDI comparison and meat equivalents.

Nutrient	Content per 100 g	% RDI	Equivalent in Meat (100 g)	References
Moisture	85–95 g	-	65–75 g (beef/chicken)	[16,17]
Protein (fresh weight) ^1^	1.9–3.3 g	3.8–6.6	20–26 g (beef/chicken)	[17,18,19]
Fat	0.2–0.6 g	-	5–20 g (beef/chicken)	[16,17]
Ash	0.85 g	-	1.0–1.5 g	[16,17]
Carbohydrate	3–7 g	1–2.3	0 g (beef/chicken)	[17,18,19]
Dietary fiber	1–2 g	4–8	0 g (beef/chicken)	[17,18,19]
Total sugars	1–2 g	-	0 g (beef/chicken)	[16,17]
Energy	22–37 kcal	1.1–1.9	250–300 kcal (beef/chicken)	[17,18,19]
Ergosterol	56 mg	-	0 g (beef/chicken)	[16,17]
Calcium	2–6 mg	0.2–0.6	5–12 mg	[17,19,20]
Copper, Cu	0.1–0.5 mg	11–56	0.08–0.15 mg	[16,17,19]
Iron	2–7 mg	11–39	2.5–3.5 mg	[16,17,19]
Magnesium, Mg	9–20 mg	2–5	20–25 mg	[16,17,19]
Manganese, Mn	0.047 mg	2	0.01–0.02 mg	[16,17,19]
Phosphorus, P	86–120 mg	12–17	200–220 mg	[16,17,19]
Potassium, K	304–420 mg	6–9	300–350 mg	[16,17,19]
Selenium, Se	2.6–26 µg	5–47	14–26 µg	[16,17,19]
Sodium, Na	5–18 mg	0.2–0.8	60–75 mg	[16,17,19]
Zinc, Zn	0.5–1 mg	5–9	4–6 mg	[17,19,21]
Vitamin C, total ascorbic acid	0.0–2.1 mg	0–2.3	0 mg (beef/chicken)	[17,19,21]
Thaimin (vitamin B_1_)	0.081–0.1 mg	6.8–8.3	0.06–0.08 mg	[17,19,21]
Riboflavin (vitamin B_2_)	0.2–0.5 mg	15–38	0.2–0.3 mg	[17,19,21]
Niacin (vitamin B_3_)	3.6–4.9 mg	23–31	5–8 mg	[17,19,21]
Vitamin B_6_	0.1–0.3 mg	6–18	0.4–0.6 mg	[17,19,21]
Vitamin B_12_	0.04 µg	1.7	2.4–2.6 µg	[17,19,21]
Pantothenic acid (vitamin B_5_)	1.50 mg	30	0.6–0.8 mg	[17,19,21]
Folate (vitamin B_9_)	17–38 µg	4.3–9.5	6–20 µg	[17,19,21]
Vitamin E (alpha-tocopherol)	0.01 µg	0.07	0.6–0.8 mg	[17,19,21]
Vitamin D	<40–1200 * IU	7–800	0–2 IU	[17,19,21]
**Essential Amino Acids ^2^ (mg/g protein, dry weight basis)**				
Histidine (His)	22–29	Meets FAO/WHO	28–35 ^3^	[8,15]
Isoleucine (Ile)	38–50	Meets FAO/WHO	48–55	[8,15]
Leucine (Leu)	60–80	Meets FAO/WHO	78–85	[8,15]
Lysine (Lys)	48–65	Meets FAO/WHO	85–95	[8,15]
Methionine + Cysteine [22]	18–26	Meets FAO/WHO	35–42	[8,15]
Phenylalanine + Tyrosine [22]	45–62	Meets FAO/WHO	72–80	[8,15]
Threonine (Thr)	36–50	Meets FAO/WHO	42–50	[8,15]
Tryptophan (Trp)	9–15	Meets FAO/WHO	12–15	[8,15]
Valine (Val)	44–58	Meets FAO/WHO	50–58	[8,15]

^1^ * Lower values (<40 IU) for cultivated mushrooms grown in dark conditions; higher values (up to 1200 IU) for wild varieties or UV-exposed mushrooms. Protein content presented here reflects fresh weight basis (1.9–3.3 g/100 g). On dry weight basis, protein content of commercially cultivated edible mushroom species ranges from 19 to 35%, with complete essential amino acid profiles, including all indispensable amino acids (lysine, leucine, isoleucine, valine, threonine, phenylalanine, methionine, tryptophan, and histidine) and protein digestibility rates of 60–80%; values are comparable to many animal-derived protein sources [15]. ^2^ Values represent typical ranges across common edible mushroom varieties, which may vary based on growing conditions, maturity, and storage. ^3^ Meat values represent average content in lean beef and chicken breast.

**Table 2:** Comparative nutritional analysis of wild edible mushroom species with superior micronutrient content (Source: [29]).

Sl No.	Scientific Name	Energy (Kcal/ 100 g)	Protein (g/ 100 g)	Carbohydrate (g/ 100 g)	Fat (g/100 g)	Dietary Fiber (g/ 100 g)	β-Carotene (μg/ 100 g)	Vit C (mg/ 100 g)	Vit B_1_ (mg/ 100 g)	Vit B_2_ (mg/ 100 g)	Total Folate (μg/ 100 g)	Iron (mg/ 100 g)	Zinc (mg/ 100 g)	Calcium (mg/ 100 g)
1.	*Termitomyces fuliginosus*	41.5	2.5	7.5	0.2	6.1	9.4	<1.25	1.7	0.5	2.9	6.6	0.9	11.2
2.	*Russula c.f kanadii*	28.84	4.19	3.02	<0.1	1.2	<200	<1.25	<0.2	<0.2	1.9	12.1	3.5	39.3
3.	*Termitomyces microcarpus*	66.76	3.52	13.2	<0.1	6.9	9.3	<1.25	0.2	0.2	2.9	10.8	0.6	9.6
4.	*Volvariella volvacea*	34.68	4.27	4.4	<0.1	1.7	<200	1.4	2.9	0.8	0.3	3.7	1.7	34.9
5.	*Astraeus odoratus*	138	4.8	29.5	0.06	7.3	<0.01	<1.25	0.6	0.4	0.3	6.8	3.1	193.4
6.	*Astraeus asiaticus*	141	4.3	30.9	0.02	7.6	<0.01	<1.25	1.9	0.1	0.3	3	3.3	185.6
7.	*Termitomyces* *Indiud* (B)	38.35	2.2	6.9	0.2	4.9	<5.0	<1.25	1.5	0.3	5.2	4.1	0.4	4.9
8.	*Lactarius rajma* halensis	77.1	2.7	16.6	<0.1	2.4	<200	<1.25	2.2	0.6	1.9	6.5	4.1	18.5

Note: All nutritional values are on a fresh weight basis. On a dry weight basis, the protein content of wild edible mushroom species is substantially higher, with *Volvariella volvacea* reported at 19.40% dw [39], which is comparable to major commercially cultivated varieties (19–35% dw). Species were selected based on documented exceptional nutritional attributes from peer-reviewed nutritional surveys, including elevated protein content, superior micronutrient profiles, and high dietary fiber content.

**Table 3:** Techno-functional properties of mushroom powder and their relevance to innovative food product development.

Property	Description	Correlation with Product Development	References
Water Holding Capacity (WHC)	The ability to retain moisture. Ranging from 2.5 to 6.0 g water/g, due to hydrogen bonding and physical entrapment by high fiber content, particularly β-glucans and chitin.	Crucial for maintaining product texture and preventing moisture loss during processing.	[42]
Oil Holding Capacity (OHC)	The ability to absorb and retain lipids. Values ranging from 1.5 to 4.2 g oil/g protein. This can be enhanced by treatments like ultrasonication or enzymatic modification that expose hydrophobic amino acid residues.	Essential for texture enhancement and the retention of flavor compounds in food formulations.	[43]
Emulsification and Foaming Properties	Due to its amphiphilic nature, mushroom protein can stabilize oil–water interfaces and create foams. Emulsification activity often exceeds 50 m^2^/g, with emulsion stability indices over 65%. Foaming capacity ranges from 82.5 to 235%, but foam stability is variable (7–162%).	Essential for stabilizing products like dressings, sauces, and aerated foods. Emulsion and foam stability are more critical for commercial viability and extended shelf-life.	[44]
Gelation Properties	The ability to form a three-dimensional network that entraps water and other components. The minimum concentration required varies significantly by species (e.g., *Pleurotus ostreatus* requires 2%, while *Auricularia auricula* needs 18%). Gelation is influenced by heat, pH, and ionic strength.	Allows us to create desirable gel textures and mouthfeel in various food products, such as meat analogs or thickeners.	[45]
Textural Properties	Determined by the cellular structure and components like chitin and β-glucans. Fresh firmness values typically range from 2 to 15 N. Species like *Pleurotus eryngii* (8–12 N) are firmer than *Agaricus bisporus* (3–7 N).	Contributes to the meat-like mouthfeel that is valuable in plant-based meat substitutes. Processing methods, such as freeze-drying, can help to preserve texture.	[46]

**Table 4:** Correlation between physicochemical properties and functionality of mushroom powder and their relevance to innovative food product development.

Property	Description	Correlation with Product Development	References
pH and Acidity	The pH of fresh mushrooms typically ranges from 5.5 to 7.5. Species like *Agaricus bisporus* are around pH 6.2–6.5, while *Pleurotus* species are closer to 6.5–7.0.	The pH significantly affects the functional properties of mushroom proteins, with optimal emulsification and foaming often occurring in alkaline conditions (pH 10) and gelation enhanced at a neutral or slightly acidic pH.	[54]
Moisture Content and Water Activity (a_w_)	Mushrooms have 85–95% moisture and a high a_w_ of 0.95–0.98. The optimal moisture content for dried products is 8–12%.	High a_w_ in fresh mushrooms necessitates careful handling and preservation to prevent microbial growth. Controlled dehydration to the optimal moisture content is crucial for product stability and rehydration capacity.	[55]
Surface Properties	The surface hydrophobicity of mushroom proteins, which can be measured with probes like ANS, influences their functional behavior. This property can be enhanced by specific processing treatments. Isoelectric points for most mushroom proteins are typically between pH 4–5.	Directly affects protein solubility and their ability to stabilize emulsions and foams, making it a key factor in developing functional food ingredients.	[56]
Thermal Properties	The thermal transition temperature of mushroom proteins (protein denaturation) occurs between 60 and 85 °C. Polysaccharides, like β-glucans, are more thermally stable and can maintain their structure up to 120 °C.	Essential for optimizing heat processing to maintain functional properties and texture. Processing temperatures can be adjusted to either preserve or modify protein and polysaccharide structures for desired outcomes.	[56]

**Table 5:** Overview of commercially available mushroom- and mycelium-based innovative food products.

Company	Country	Products
Quorn Foods Ltd.	United Kingdom	Quorn mince, sausages, burgers, chicken-style pieces, ready meals, vegan range with potato protein binder
Four Sigmatic ^®^	United States	Focus Ground Coffee (Lion’s Mane), Mushroom Coffee Mix, Adaptogen Coffee, Chaga Elixir, Reishi Elixir
Popadelics	United States	Shiitake mushroom chips (various flavors), vacuum-fried specialty varieties
Host Defense (Fungi Perfecti)	United States	Lion’s mane capsules, Turkey Tail extract, Reishi tinctures, cordyceps supplements, multi-mushroom blends
Libre Foods S.L	Spain	Mycelium-based bacon alternative: Libre Bacon (oyster mushroom and pea protein)
Mush Foods, Ltd.	Israel	Mycelium ingredient for hybrid meat products: 50CUT-mycelium blended with half meat in hybrid burgers
Fungi Perfecti, LLC. (Host Defense^®^)	United States	Mycelium-based functional drink mixes: MycoBrew^®^: coffee, matcha, cocoa, and mocha blends with lion’s mane mycelium
Mud\Wtr	United States	Mushroom-based coffee alternative: matcha chai blend with cordyceps, lion’s mane, chaga, Reishi
Spacegoods	United Kingdom	Adaptogenic powder drink mixes with mushrooms. Rainbow Dust with lion’s mane, cordyceps, chaga
Ppuff!	Indonesia	Crisp mushroom snacks: Snacks made from red rice, corn, and mushrooms
Rritual Superfoods	Canada	Offers a range of mushroom-based functional powders, rather than ready-to-eat meals: Chaga Immune, Reishi Relax, Lion’s Mane Focus
Life Cykel	Australia	Life Cykel specializes in double liquid mushroom extracts crafted from both fruiting bodies and mycelium: Lion’s mane extract, cordyceps extract, Reishi extract, chaga extract, Turkey Tail extract, shiitake extract

Note: Products are derived from fungal mycelium, rather than mushroom fruiting bodies. Mycelium and fruiting bodies differ substantially in β-glucan linkage patterns, protein content, and bioactive compound profiles; nutritional and functional equivalence cannot be assumed.

**Table 6:** Recent developments in mushroom-based innovative snack and food products: a critical review of formulations, technologies, and nutritional outcomes.

Innovative Product	Species Used	Preparation/Technology	Nutritional Highlights/Physiological Effects or Health Claims	References
Flatbread (chapatti)	*Pleurotus* spp. or *Agaricus bisporus* as dried mushroom powder	D-optimal mixture design; mushroom drying and powdering; blending with wheat and millet flours; dough rheology, texture and SEM analysis	Increased protein, fiber, ash, vitamin D_2_ (3812 IU/100 g) and antioxidant activity; reduced chapatti hardness; improved functional value	[69]
Soy–Mushroom Analog Burger	*Pleurotus ostreatus*	Full fat soy (FFS) or isolated soy protein (ISP) mixed with oyster mushroom (0–12%); texturized using twin-screw extruder with cooling die	Enhanced textural properties, improved water holding capacity and cooking properties; FFS-based with 12% mushroom showed highest quality parameters and organoleptic properties	[70]
Mushroom–Chicken Burger	*Oyster mushroom stalk*	Chicken breast meat partially substituted with oyster mushroom stalk powder (2.5–10%); formed into patties	Improved water-binding capacity, reduced cooking loss and shrinkage, enhanced antioxidant properties, increased fiber and ash content; optimal substitution at 2.5–5% level	[71]
Mushroom Nuggets	-	Utilizes mycelium for fibrous texture; shaped and breaded before baking or frying	Rich in protein, fiber; zero cholesterol; supports immune health	[72]
Mushroom-Based Sausages	*Lentinus edodes*, *Coprinus comatus* and *Pleurotus ostreatus*	Blended mushroom powder with pea protein, barley, and spices; extruded or stuffed into casings	Resulted with high in protein, low in fat, good umami profile; contains dietary fiber and essential amino acids	[73]
Commercial Plant/Mushroom Jerky	*Shiitake* and *king oyster*	Commercially produced jerky alternatives using mushroom-based formulations	Lower protein content (3 g/serving) compared to plant-based alternatives; consumer acceptance challenged by excessive toughness and chewiness; optimal pricing below current market rates	[74]
**Processed/Preserved Products**				
Reduced-Sulfite Canned Mushrooms	*Agaricus bisporus*	Canned mushrooms with controlled/reduced sulfite content, using alternative preservation methods and condiments	Maintained stable pH (<4.5) for 42 days without sulfites; “sulfite-free” labeling increased consumer purchase intention despite slight color changes; commercially viable with appropriate marketing	[75]
**Snack Products**				
Mushroom Chips	*Agaricus bisporus*	Thinly sliced mushrooms (*shiitake*, *portobello*) dehydrated or vacuum-fried/seasoned	Gluten-free, low calorie and fat; contains antioxidants and B vitamins	[76]
Mushroom Crackers	*Pleurotus ostreatus*	Mushroom powder incorporated into dough; baked	Higher protein and lower carbohydrates than conventional crackers; fiber-rich	[77]
Mushroom-Based Snack Bars	Lentinula edodes	Mixed mushroom powders with seeds, nuts, grains; pressed and packaged	Rich in bioactive compounds (protocatechuic, p-hydroxybenzoic, p-coumaric and cinnamic acids), proteins, and fibers; energy-boosting	[78]
**Beverages**				
Mushroom-Infused Tea	*Pleurotus sajor-caju*	Infusion of dried mushroom (e.g., Reishi, lion’s mane) with herbs	Provides adaptogens, immune-boosting polysaccharides, low-calorie	[79]
Mushroom Coffee	*Hericium erinaceus* and *cordyceps militaris*	Coffee blended with powdered mushrooms (e.g., lion’s mane, chaga)	Offers caffeine + cognitive benefits (hericenones, erinacines); reduces anxiety, supports focus	[80]
Shiitake Beverage with Quality Control	*Shiitake*	Beverage formulation with quantified polysaccharide content, using NIR spectroscopy and machine learning for quality assurance	Standardized polysaccharide content with rapid, non-destructive quality control; enables consistent bioactive compound delivery	[81]
Mushroom-Enhanced Fermented Wine	*Pleurotus pulmonarius*	Co-fermentation using mushroom mycelia and yeast (42:58 ratio) at optimized conditions (pH 4.99, 28.29 °C, 131 h)	Enhanced ethanol production (22.29% vs. conventional 13–14%), increased antioxidant activity, extends shelf-life of perishable fruits	[82]

**Table 7:** Advances in food processing technologies and associated innovations: critical evaluation of applications, advantages, limitations, and recent achievements in mushroom-based product development.

Technology	Application	Advantages	Limitations	Recent Achievement	Reference
High Moisture Extrusion	Meat alternatives	Made fibrous, meat-like texture	High energy consumption, limited to specific moisture content ranges (45–75%)	Temperature-controlled processing (120–180 °C)	[99]
Fermentation Technology	Mycoprotein production	Improved in digestibility and flavor	Extended processing time (days to weeks), contamination risk, pH sensitivity	Continuous fermentation systems, pH control	[129,130]
Freeze Drying	For preservation and snack development	Maintains nutritional quality, avoids aroma loss and/or oxidation associated with conventional drying	Extremely high energy costs, long processing times (24–48 h), equipment intensive, high investment cost	Hybrid and atmospheric freeze-drying systems reduce energy use by up to 30% and drying time by up to 70%; integration with microwave, infrared, or ultrasound technologies enhances efficiency and preserves quality	[131]
High-Pressure Processing	Safety, shelf-life extension	Non-thermal preservation	High capital investment, limited to liquid/semi-solid products, texture changes in some foods	400–600 MPa pressure applications	[132]
Ultrasonic Extraction	To extract bioactive compounds	Improved extraction efficiency	Equipment fouling, limited penetration depth, potential compound degradation at high intensities	Optimization of frequency and time parameters	[133]
3D Food Printing	Novel textures, customization	Personalized nutrition, reduced waste	Limited material compatibility, slow processing speed, high initial costs, scalability issue	Integration with mushroom-based gels and pastes	[134]

## Data Availability

No new data were created or analyzed in this study.
